# *Intra*fractional 6D head movement increases with time of mask fixation during stereotactic intracranial RT-sessions

**DOI:** 10.1186/s13014-019-1425-7

**Published:** 2019-12-18

**Authors:** Julian Mangesius, Thomas Seppi, Rocco Weigel, Christoph Reinhold Arnold, Danijela Vasiljevic, Georg Goebel, Peter Lukas, Ute Ganswindt, Meinhard Nevinny-Stickel

**Affiliations:** 1 Department of Therapeutic Radiology and Oncology, Medical University of Innsbruck, Anichstraße 35, A-6020 Innsbruck, Austria; 2 Department of Medical Statistics, Informatics and Health Economics, Medical University of Innsbruck, Innsbruck, Austria

**Keywords:** Frameless stereotactic radiation therapy, Thermoplastic mask head fixation, Intrafractional accuracy, X-ray position monitoring, ExacTrac

## Abstract

**Background:**

The present study investigates the intrafractional accuracy of a frameless thermoplastic mask used for head immobilization during stereotactic radiotherapy. Non-invasive masks cannot completely prohibit head movements. Previous studies attempted to estimate the magnitude of intrafractional inaccuracy by means of pre- and postfractional measurements only. However, this might not be sufficient to accurately map also intrafractional head movements.

**Materials and methods:**

Intrafractional deviation of mask-fixed head positions was measured in five patients during a total of 94 fractions by means of close-meshed repeated ExacTrac measurements (every 1.4 min) conducted during the entire treatment session. A median of six (range: 4 to 11) measurements were recorded per fraction, delivering a dataset of 453 measurements.

**Results:**

Random errors (SD) for the x, y and z axes were 0.27 mm, 0.29 mm and 0.29 mm, respectively. Median 3D deviation was 0.29 mm. Of all 3D intrafractional motions, 5.5 and 0.4% exceeded 1 mm and 2 mm, respectively. A moderate correlation between treatment duration and mean 3D displacement was determined (r_s_ = 0.45). Mean 3D deviation increased from 0.21 mm (SD = 0.26 mm) in the first 2 min to a maximum of 0.53 mm (SD = 0.31 mm) after 10 min of treatment time.

**Conclusion:**

Pre- and post-treatment measurement is not sufficient to adequately determine the range of intrafractional head motion. Thermoplastic masks provide both reliable interfractional and intrafractional immobilization for image-guided stereotactic hypofractionated radiotherapy. Greater positioning accuracy may be obtained by reducing treatment duration (< 6 min) and applying intrafractional correction.

**Trial registration:**

Clinicaltrials.gov, NCT03896555, Registered 01 April 2019 - retrospectively registered.

## Background

In recent years, advances in non-invasive patient immobilization as well as in image-guided radiation therapy (IGRT) have enabled the use of thermoplastic masks and hypofractionated radiotherapy for single brain metastases [1].

The use of non-invasive thermoplastic masks allows for fractionated RT, thereby overcoming the main limitation of invasive head fixation [2, 3]. Several studies have shown that image guidance makes set-up and repositioning uncertainty with the non-invasive mask immobilization comparable to that of invasive stereotactic ring application [2, 4–6]. Nevertheless, this method may have less intrafractional accuracy due to the non-rigid construction, indirect immobilization of the skull, and unpredictable patient movement. Many studies reported this effect only by means of quantifying pre- and postfractional deviations of the patient’s head by either CBCT (cone beam computed tomography) or ExacTrac [2, 4, 7, 8]. However, real intrafractional movements cannot be mapped by measuring the position of the head only at the beginning and the end of treatment since this gives no information on possible head movements during the individual irradiation treatments. Larger deviations would need to be accounted for by increasing the PTV margins, thereby exponentially increasing the irradiated volume and the risk of complications, such as radionecrosis [9–11]. Especially, novel single-isocenter intracranial irradiation techniques for multiple metastases [12–14] demand for highest precision since even smallest-scale rotational deviations may lead to insufficient target coverage of more distant lesions.

The purpose of the present study was to evaluate the precision and reliability of mask fixation of the head during the entire duration of stereotactic RT sessions. In order to assess position accuracy not only at the beginning and the end of the sessions, we repeatedly mapped deviations of the head position in both translation and rotation, by concomitantly measuring intrafractional movement using the ExacTrac 6D X-Ray Positioning System (Brainlab AG, Munich, Germany). From the obtained data we evaluated the need to adjust safety margins around the gross tumor volume (GTV).

## Materials and methods

### Patients and inclusion criteria

Intrafractional variations were evaluated in a non-randomized group of five patients (Additional file 1: Table S1) during N_F_ = 96 treatment sessions with a total of *N* = 551 ExacTrac measurements. ExacTrac imaging was used multiple times to monitor intrafractional movements of the head during beam-on time of single sessions. Intrafractional measurements were not used to correct patient’s head position during the RT session. Corrections were performed only once up-front, as is standard practice at our clinics.

The study involved patients who had a single intracranial tumor or metastasis. Linear accelerator-based stereotactic image-guided radiotherapy was administered between November 2014 and September 2015. Two patients were treated with a hypofractionated regimen (five fractions), whereas three patients were treated according to a conventionally fractionated schedule (30 fractions). Immobilization was performed with the Brainlab Thermoplastic Mask (Brainlab AG, Munich, Germany). To ensure patient compliance and provide a homogeneous study population all prospectively selected patients were required to have a Karnofsky Performance Score (KPS) greater than 80% as well as good cooperation capability. Treatment planning and course were identical for conventional and hypofractionated treatments (LinAc Versa HD, Elekta AB, Stockholm, Sweden). Target volume definition was performed on fused planning CT and contrast enhanced t1 weighted MRI images using Brainlab iPlan RT Image (v4.5.3; Brainlab AG, Munich, Germany). Treatment planning was performed with Brainlab iPlan RT Dose (v4.5.3) as well as Pinnacle (v9.8; Philips Medicals, Fitchburg, WI, USA).

### Clinical workflow and intrafractional measurements

To detect intrafractional motion during treatment delivery, the ExacTrac in-room based monitoring system (Brainlab AG, Munich, Germany) was used as previously described [15, 16]. It was employed in this study to repeatedly record 3D deviations of the target isocenter for both translation and rotation, during a session of dose application that lasted up to 20 min (workflow shown in Additional file 1:Figure S1).

Following thermoplastic mask molding, contrast-enhanced treatment planning CT was performed with a reconstructed slice thickness of 1.5 mm. CT scans were also used for image registration to reference ExacTrac recordings and CBCT-guided patient positioning at the beginning of each treatment session.

Calculated 6D shifts were checked and, if indicated, translational and rotational deviations from reference positions were computed and corrected by adjusting the treatment couch (equipped with the HexaPod evo RT system, Elekta AB, Stockholm, Sweden). CBCT check was repeated until translational deviation in each direction was < 1.0 mm and rotational errors were < 1.0°. Next, the first ExacTrac measurement was taken before treatment start at a rotatable baseplate position of 0°. This initial ExacTrac recording was used as a reference point for comparison with the subsequent intrafractional measurements made during irradiation (*N* = 3 to 10). Patient positions were not corrected during a treatment session.

Treatment plans in this study comprised both converging arcs with conical collimators and multiple isocentric fields with individually shaped beams using micro-multileaf collimator. ExacTrac measurements were taken simultaneously with arc irradiations (five per fraction) at gantry angles of 0°, 90°, 180° or 270° with a tolerance range of +/− 10°. For shaped beam application, ExacTrac recordings were taken immediately after each field application (five to eight per fraction). At baseplate angles of 90° and 270° it was not possible to detect positioning since the couch-mounted metal frame used for mask fixation shields parts of the ExacTrac radiographs. A final pair of ExacTrac images at a reset baseplate position of 0° was acquired at the end of each treatment session.

### Correction of data and phantom study

At each step of the radiotherapy treatment course different sources of inaccuracy can accumulate. To correctly map the patient’s intrafractional motions depending on mask fixation, other factors potentially causing positioning errors had to be eliminated. Ideally, the linear accelerator’s gantry, collimator, and table all rotate with respect to a single point called the isocenter. In reality, unavoidable misalignments prevent the rotation axes from intersecting at a single point, but instead only near each other within a sphere [17].

The baseplate angle position was identified as a main system-inherent source of measured isocenter deviations. The deviation was largest at an angle of 50° with an average 3D vector (3DV) of 0.82 mm. Phantom measurements were performed to quantify this error for each baseplate angle used in this study in order to correct our results by considering the recorded deviations.

A second cause of mask-independent errors can be addressed to residual errors derived from initial patient positioning. The first ExacTrac measurement of every fraction was therefore used as a point of reference and mask-independent 6D deviations were subtracted from all subsequent measurements.

### Statistical analysis

Translations in the x (medial-lateral), y (superior-inferior) and z (anterior-posterior) directions as well as rotations around the x (transversal), y (longitudinal) and z (sagittal) axes were analysed. The resulting 3DV was calculated as follows:

$$ 3\mathrm{DV}=\sqrt{{\mathrm{x}}^2+{\mathrm{y}}^2+{\mathrm{z}}^2} $$

IBM SPSS Statistics 22 (IBM Cooperation, Armonk, NY, USA) was used for statistical analysis of corrected values. An independent samples *t*-test was performed to compare positioning deviations of the first five fractions with those of the subsequent ones in the group of conventionally fractionated patients. To evaluate the correlation between the time elapsed since the first measurement in each treatment session and the 3DV, Spearman’s rank correlation coefficient was calculated. An analysis of variance (ANOVA) was used to analyse this correlation further. A *p* value of < 0.05 was deemed significant.

## Results

A total of 551 ExacTrac measurements were made with thermoplastic masks during 96 fractions of facilitated positioning. Two measurements had to be excluded from statistical analysis because of failed fusion between ExacTrac radiographs and DRRs. The first measurement of each fraction was used as reference. Hence, a total of 453 positioning recordings were statistically analysed.

**Fig. 1:**
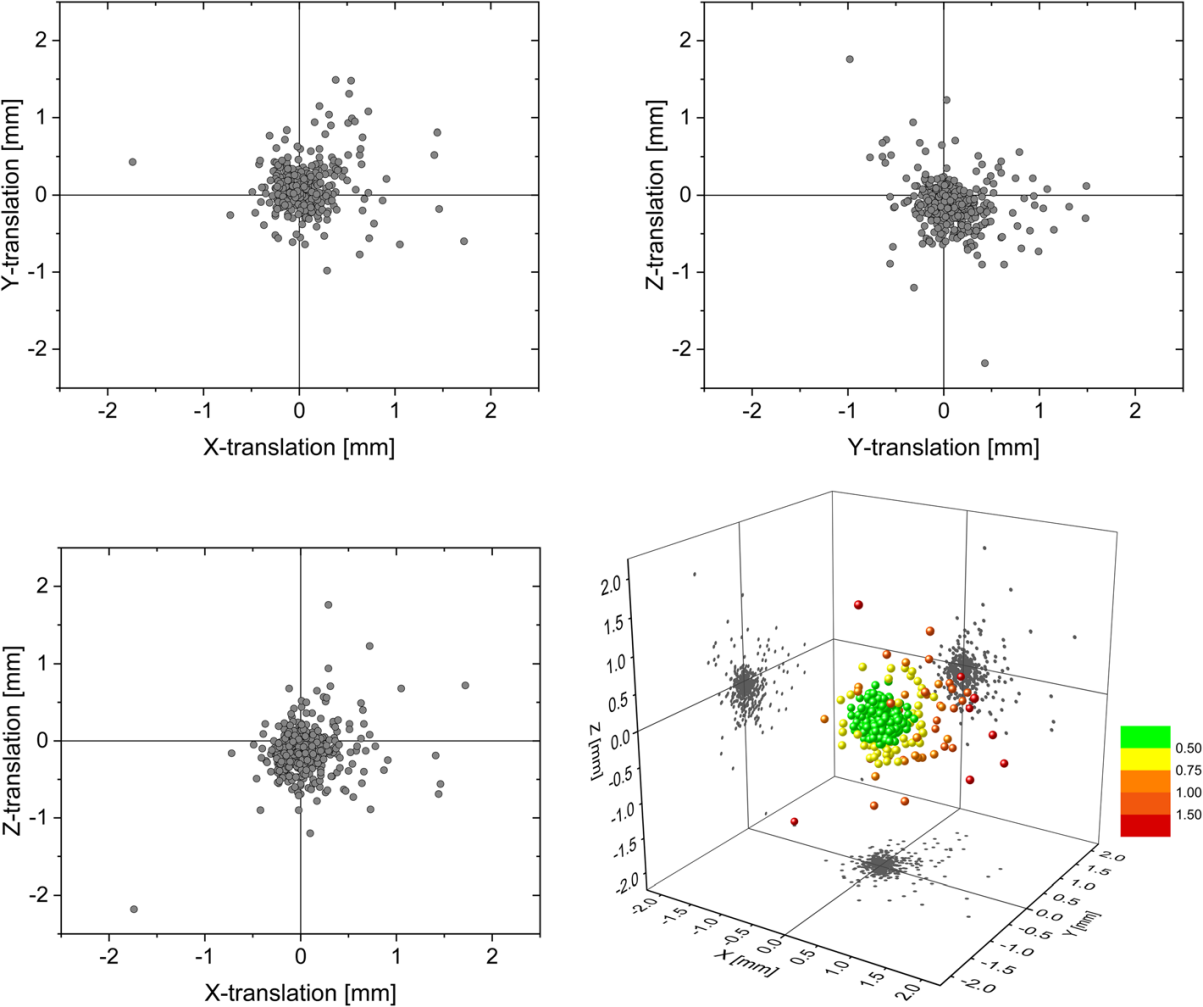
Scatterplots of intrafractional positioning deviations in the coronal plane (xy), axial plane (xz) and sagittal plane (yz) during thermoplastic mask fixation of the head

The mean number of measurements per fraction was six and ranged from four to 11. The recorded translational and rotational errors are presented in Table 1. Standard deviation (SD) of spatial displacements, used as a measure of random errors, was 0.27 mm, 0.29 mm and 0.29 mm in the x, y and z directions, respectively. SD of the corresponding rotational errors was 0.33°, 0.28° and 0.55°. Maximum spatial displacements (Fig. 1) were 1.74 mm (x axis), 1.49 mm (y axis) and 2.18 mm (z axis), and the largest rotational errors were 2.18° (x° angle), 1.52° (y° angle), and 2.27° (z° angle).

**Table 1:** Overview of translational and rotational errors with descriptive statistics of absolute deviations in head position caused by patient motion

*N* = 453 (N_F_ = 96)	3DV [mm]	Translations [mm]			Rotations [°]		
		X	Y	Z	X°	Y°	Z°
Median	0.29	0.11	0.12	0.14	0.15	0.11	0.22
Mean	0.38	0.17	0.19	0.21	0.22	0.19	0.36
95% CI							
Lower	0.35	0.15	0.17	0.19	0.19	0.17	0.32
Upper	0.41	0.19	0.21	0.23	0.24	0.21	0.40
Variance	0.11	0.05	0.05	0.05	0.06	0.04	0.17
SD	0.34	0.22	0.22	0.23	0.25	0.21	0.42
Maximum	2.82	1.74	1.49	2.18	2.81	1.52	4.13
Percentile							
95%	1.06	0.56	0.62	0.63	0.62	0.58	1.14
25%	0.16	0.04	0.05	0.06	0.07	0.05	0.09

**Fig. 2:**
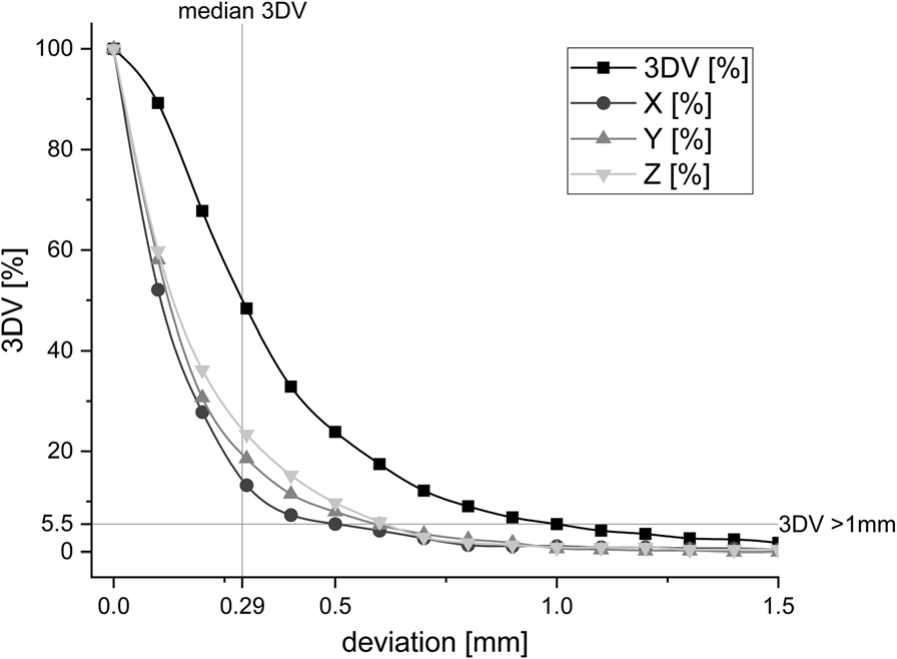
Histogram of cumulative intrafractional 3DV displacements and proportionate x (transversal), y (longitudinal) and z (sagittal) translations using a thermoplastic mask for head fixation

Mean deviation along the individual axes was close to the point of origin (< 0.10 mm), accompanied by an average rotation angle of < 0.08° for each axis. As a result, no significant systematic deviation was recorded. In absolute values, 95% of the deviations were smaller than 0.56 mm (x), 0.62 mm (y) and 0.63 mm (z). Mean 3DV deviation was 0.38 mm (SD = 0.34 mm; upper 95% CI = 0.41 mm). Of all 3D intrafractional motions 18.5, 5.5 and 0.4% exceeded 0.5 mm, 1 mm and 2 mm, respectively. A maximum 3DV error of 2.82 mm was recorded. Of the 3DV deviations 95% were smaller than 1.06 mm (Fig. 2).

**Fig. 3:**
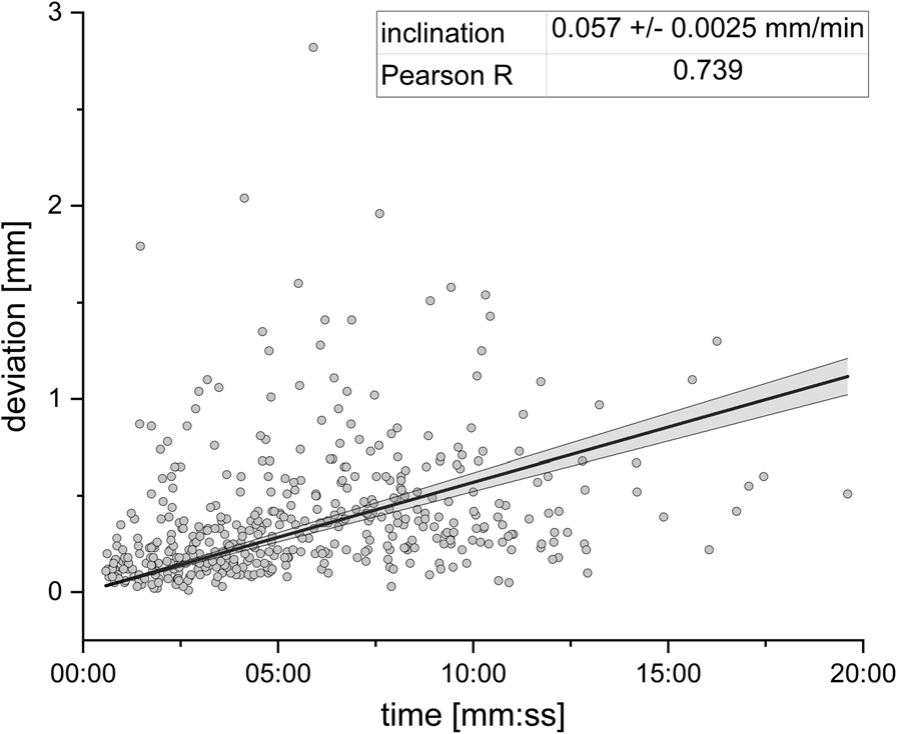
Correlation between head motion (3DV) and elapsed time of intrafractional head fixation using a thermoplastic mask

Independent samples *t*-test delivered no difference (t = 1.239, df = 391, *p* = 0.216) in intrafractional motion of conventionally fractionated patients between the first five (mean 3DV = 0.37 mm, SD = 0.32 mm) and the remaining 25 treatment sessions (mean 3DV = 0.32 mm, SD = 0.28 mm). Mean duration from the first to the last ExacTrac measurement in each treatment session was 9 min 18 s (N_F_ = 96; Min. = 4 min 29 s; Max. = 19 min 36 s). A moderate correlation between head motion (3DV) and time elapsed since the first measurement in each session was observed (Fig. 3). Spearman’s rank correlation was run to analyse this relationship, which was statistically significant (r_s_ = 0.45, *N* = 453, *p* < 0.01).

**Fig. 4:**
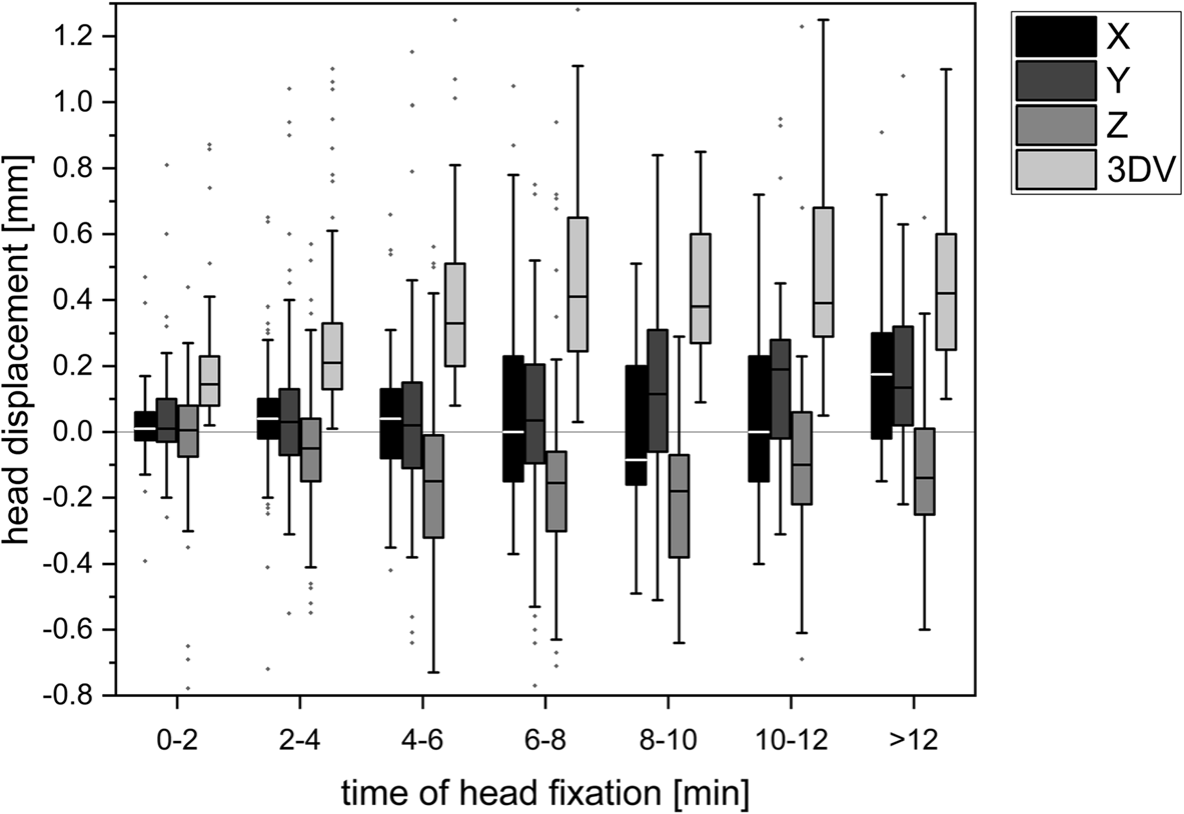
Head motion as a consequence of intrafractional treatment duration

Data points for 3DV head displacements were split into groups of consecutive 2-min intervals (Fig. 4). One-way ANOVA was run to determine whether significant differences can be observed in head motion depending on the duration of treatment. Grouped measurement results used in this test are described in Additional file 1: Table S2. Significant differences between the extent of head displacements were observed between the 2-min interval groups (F (6, 446) = 9.790, *p* < 0.001). A post hoc Games-Howell test showed a significant increase in quantified positioning deviations within the three interval groups of the first 6 min (*p* < 0.03). No significant further increase in the spatial deviation of mask-fixed head positions was observed after 8 min. Mean 3DV head displacements increased from 0.21 mm (SD = 0.26 mm) in the 0- to 2-min interval group to a maximum of 0.53 mm (SD = 0.31 mm) after 10 min of treatment time.

Time-dependent deviations were also analysed separately for each axis. One-way ANOVA showed a significant difference along the longitudinal y axis (F [6, 446] = 3.12, *p* = 0.005) and the sagittal z axis (F [6, 446] = 3.51, *p* = 0.002). Overall, discrete time-dependent systematic movements in the positive y direction and in the negative z direction were identified.

**Fig. 5:**
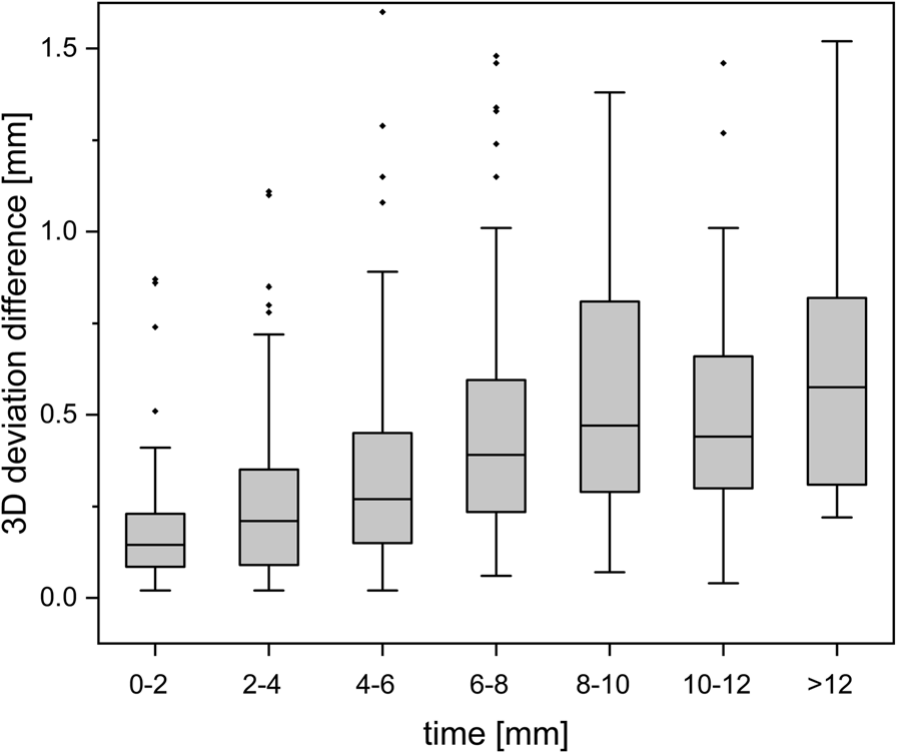
Continuous 3D head motion between individual consecutive ExacTrac measurements (_Δ_3DV) within a treatment session

In addition, the three-dimensional variance in patient head position between two consecutive ExacTrac measurements (_Δ_3DV) during an intrafractional treatment course was quantified. A continuous 3D head motion between each measurement was observed and its absolute value in spatial deviation calculated (Fig. 5). Descriptive statistics are displayed in Table 2. A significant increase in intercourse positioning variance was demonstrated with one-way ANOVA (F (7, 438) = 8.30, *p* < 0.001). Mean magnitude of continuous intrafractional patient motions increased depending on the duration of treatment. Mean _Δ_3DV assessed during the first 2 min of treatment was 0.21 mm (SD = 0.26 mm) and reached 0.66 mm (SD = 0.41 mm) after 12 min.

**Table 2:** Descriptive statistics of continuous 3D head motion between single consecutive ExacTrac measurements (_Δ_3DV) within a treatment session

Time [min]	N	_Δ_3DV Mean [mm]	SD	Confidence interval of the mean	
				Lower	Upper
0–2	68	0.21	0.26	0.15	0.27
2–4	113	0.29	0.29	0.24	0.35
4–6	87	0.39	0.42	0.30	0.48
6–8	72	0.52	0.42	0.42	0.61
8–10	58	0.61	0.53	0.47	0.75
10–12	33	0.53	0.38	0.40	0.67
> 12	22	0.66	0.41	0.47	0.84

To conclude, ExacTrac coordinates of maximum intrafractional head position deviation and the respective coordinates at the beginning and the end of fraction were compared. In 43 (45.7%) of 96 analyzed fractions, 3D deviation did not exceed 0.5 mm - either during intrafractional measurements or at the end of the treatment session – as compared to the initial head position at treatment start. In 26 (27.7%) of 94 fractions at least one intrafractional measurement showed a deviation larger than 0.5 mm. However final ExacTrac measurement at the end of the session again revealed a 3D deviation of less than 0.5 mm. Finally, in 18 (19.1%) of 94 fractions, a deviation of more than 0.5 mm in mask-fixed head position was found both during the session and after completion of RT.

## Discussion

The high radiation doses used and the proximity to vital structures make accurate positioning and precise irradiation of utmost importance in the radio-surgical treatment of intracranial metastases. The use of thermoplastic masks offers substantial advantages concerning ease of use and patient comfort. In IGRT, the repositioning accuracy of thermoplastic masks, as compared to that of frame-based stereotactic head fixation, has been demonstrated in several studies to be equal (< 1 mm) [2, 4, 5, 18].

Kataria et al. [19] investigated positioning accuracy of a thermoplastic mask using pre- and post-fractional imaging in six patients. The mask’s mean displacements at the end of the treatment sessions were reported to be 0.60 mm (SD = 1.80 mm), 0.20 mm (SD = 0.60 mm) and 0.00 mm (SD = 0.50 mm) in the x, y and z directions, respectively. In a similar study Ramakrishna et al. [2] reported data obtained from 110 stereotactic radiotherapy sessions and found a mean 3D deviation of 0.7 mm (SD = 0.5 mm). In 22% of all sessions a 3D displacement of larger than 1 mm was found. Linthout et al. [7] published data obtained from 385 pre- and postfractional stereoscopic x-ray images. The mean translations were 0.0 mm (SD = 0.7 mm), 0.3 mm (SD = 0.7 mm) and − 0.5 mm (SD = 1.2 mm) in the three directions, and mean rotational errors were − 0.2° (SD = 0.8°), 0.1° (SD = 0.7°) and − 0.1° (SD = 0.6°). Lamba et al. [4] found translational deviations of 0.1 mm (SD = 0.3 mm), − 0.1 mm (SD = 0.5 mm) and 0.1 mm (SD = 0.3 mm). Of the measurements 6.5% exceeded 1 mm in any direction.

All of these studies measured the ‘intra’fractional deviations by comparing only pre- and post-fractional measurements from CBCT or stereoscopic x ray imaging. However, this procedure is not suitable for determining the loss of accuracy caused by intrafractional head motions during an individual treatment session, because the magnitude, direction and angle of the motions do not necessarily remain constant over the course of treatment.

In the present study we measured the intrafractional accuracy by making repeated ExacTrac measurements per session in a non-randomized group of five patients delivering a dataset of 453 measurements recorded during 96 treatment sessions.

Random positioning errors assessed in our study show overall smaller intrafractional head displacements than in the previously described studies. Random errors (SD) in the translational deviations did not exceed 0.29 mm in any direction. The largest random rotational error (SD) was 0.55°. The mean 3DV length was 0.38 mm. No relevant systematic deviation was observed. By contrast to others, only 5.5% of all 3DV measurements exceeded 1 mm.

Accounting for only the first and last ExacTrac measurement of each investigated fraction in our study would result in an increase to 0.49 mm in calculated mean intrafractional 3D error (+ 29.2%) versus the mean 3DV length of 0.38 mm determined from four to 11 measurements per session. Studies using only pre- and post-fractional position measurements therefore tend to overestimate the influence of intrafractional motions on factual treatment accuracy impairment.

A possible explanation for these contradicting findings might be our finding that the magnitude of motion continuously increases with time during radiotherapy sessions. In addition, the time interval between pre- and postfractional measurements in the discussed studies was substantially longer (15 min to even exceeding 30 min in some cases) than in our study, which showed a mean time interval of repeated intrafractional measurements of 1.5 min.

The relevance of real intrafractional head motion is further underlined by our finding that during 27.7% of all investigated fractions intrafractional motion of more than 0.5 mm was detected at least once during beam delivery, whereas the final ExacTrac measurement at the end of these sessions revealed a 3D deviation of less than 0.5 mm.

Badakhshi et al. [20] examined intrafractional motions in a prospective cohort of patients during 269 stereotactic radiosurgery sessions using ExacTrac measurements after each new table position (3.6 measurements per fraction). Mean SD of all translations in any direction was reported as 0.8 mm. Mean 3DV was 1.05 mm (SD = 0.93 mm). Displacement errors were corrected when exceeding a value of 0.7 mm or 1°. Despite this measure, still 37% of all 3D deviations exceeded 1.0 mm as compared to 5.5% as reported by us. The timeframe between measurements (~ 4.2 min) was also markedly longer than that applied in our study, thus potentially helping explain the substantial differences in positioning accuracy, even though the same mask system was used.

In contradiction to Lewis et al. [21], we detected a moderate correlation (r_s_ = 0.45) between time elapsed since setup and observed 3DV length. Within the first 6 min of treatment, a steep increase in 3D translational errors from 0.21 mm (SD = 0.26 mm) to 0.51 mm (SD = 0.35 mm) was observed, reaching a plateau after 8 to 10 min. These results are analogous to those reported by Amelio et al. [22], reflecting the influence of the time interval between pre- and postfractional measurements on intrafractional positioning accuracy. The authors assumed that patients might start to relax after a certain adaptation period, thereby causing the intrafractional displacements to reach a plateau.

However, this hypothesis contradicts our findings, which clearly show that the magnitude of individual intrafractional motions (_Δ_3DV) increased continuously along with each consecutive ExacTrac measurement during treatment. Although patient restlessness continuously increases during a treatment session, the thermoplastic mask’s semi-flexible material restricts spatial displacements of the isocenter to a certain range until maximum freedom of movement has been reached.

This might explain why the magnitude of new movements still increases over time, without causing an increase in total positional displacement. Our finding is supported by Wang et al. [23], who also noted a time-dependent increase in 3DV length from 0.34 mm to 0.77 mm within a time-frame of 45 min during 50 radiosurgery sessions. Along with our own results, decreasing the duration of RT sessions correlates with less intrafractional positional displacement, thus increasing overall intrafractional treatment accuracy.

The present study focused on the technical aspects of the feasibility to perform intrafractional X-ray based position monitoring, and aimed to evaluate the need for additional corrections during beam-on time of a single RT session. As such, the total number of anew patient positionings (96 fractions) as well as the number of position measurements (*n* = 453) is more decisive than the total number of patients. The small sample size is a limitation of this study and might impair the generalisability of our results. Therefore future investigations including a larger patient cohort will be necessary for statistically robust analysis of the frequency, time-course, and extent of intrafractional motions.

To minimise inter-patient variability the included patients were selected for good general condition and cooperation. In fact, the study was designed primarily to demonstrate the additional inaccuracy caused by intrafractional motions, excluding all other errors that might contribute to the determination of safety margins. Such error sources might differ between institutions, mainly depending on the applied RT and head fixation technique, as well as on available treatment devices. These errors include treatment machine related uncertainties, patient (re)positioning inaccuracy, imaging related limitations for treatment planning and for image guidance, as well as target definition and treatment planning system uncertainties [24]. The fact that 5% of measurements showed head displacements exceeding 1 mm - after exclusion of all other errors except for patient motion - justifies a more in-depth discussion on the necessity of intrafractional corrections. In addition, even the most cooperative patients selected for this study exhibited a time-dependent increase in head motion, finally surpassing tolerance after 6 to 8 min of uncorrected head fixation.

To summarise, measuring the positional variation, not only before but also during beam-on time of arc radiation therapy, allows determining the exact position error also during treatment. However, position corrections upon head displacements exceeding tolerance during the beam-on time were not performed in this study, since ExacTrac imaging had to be manually triggered, and 6D displacements were recorded for later analysis only. In order to benefit from the evidenced feasibility of intra-beam position surveillance, it would therefore be essential also to implement automated beam-hold, subsequent position correction, and precise resumption of LinAc-based irradiation.

## Conclusions

Spatial displacements of the head during administration of stereotactic radiotherapy measured in this study for frameless head fixation are substantially smaller than reported by others. Safety margins of 1 mm were seen to be appropriate to account for at least 94.5% of 453 evaluations of intrafractional head positions in this highly selected group of five patients, confirming that thermoplastic masks provide adequately precise and reliable inter- and intrafractional immobilization for image-guided stereotactic radiotherapy.

To especially examine potential intrafractional head displacements, pre- and postfractional imaging alone is not suitable. For this purpose repeated intrafractional ExacTrac measurements were performed, showing that head motion increases depending on the duration of treatment. Consequently, greater intrafractional accuracy is achieved by reducing the duration of RT sessions. Alternatively, repeated verification of head position might enable intrafraction corrections in routine stereotactic RT, best applicable if supported by an automated beam-hold system in future practice.

## Additional file

**Additional file 1: Table S1.** Patient and treatment characteristics. **Table S2.** Descriptive statistics of head motion (3DV), as well as of x, y and z displacements, grouped as measurement cohorts collected within 2-min intervals after treatment start. **Figure S1.** Patient setup and repeated intrafractional tracking of head movement.

## Data Availability

The datasets used and analysed during the current study are available from the corresponding author on reasonable request.

## Abbreviations

3DV: 3D vector
ANOVA: Analysis of variance
CBCT: Cone beam computed tomography
CI: Confidence interval
IGRT: Image-guided radiotherapy
KPS: Karnofsky Performance Scale
r_s_: Spearman’s rank correlation coefficient
RT: Radiotherapy
SD: Standard deviation
_Δ_3DV: Change in 3D vector between two consecutive measurements
