# Supplementary material for: Intrafractional 6D head movement increases with time of mask fixation during stereotactic intracranial RT-sessions
Source: Radiat Oncol. 2019 Dec 18;14:231. doi: 10.1186/s13014-019-1425-7 (PMC6921566; doi:10.1186/s13014-019-1425-7)
Supplement: Supplementary file 1 — Additional file 1: Table S1. Patient and treatment characteristics. Table S2. Descriptive statistics of head motion (3DV), as well as of x, y and z displacements, grouped as measurement cohorts collected within 2-min intervals after treatment start. Figure S1. Patient setup and repeated intrafractional tracking of head movement. [file 13014_2019_1425_MOESM1_ESM.pdf]

Supplementary Table 1: Patient and treatment characteristics

		Total (n = 5)
Age (y)	Median	52
	Range	32 – 72
Sex	Female	3
	Male	2
BMI	Mean (SD)	28.1 (5.85)
	Range	22.4 – 38.6
KPS	100	2
	90	2
	80	1
Tumor entity	Lung cancer metastasis	2
	Pituitary adenoma	2
	Glomus hypothympanicum tumor	1
PTV	Range [cm^3] (Mean)	6.56 – 69.91 (22.0)
GTV	Range [cm^3] (Mean)	0.88 – 22.30 (8.7)

Supplementary Table 2: Descriptive statistics of head motion (3DV), as well as of x, y and z displacements, grouped as measurement cohorts collected within 2-minute intervals after treatment start.

Time [min]	Axis	N	Mean [mm]	SD	Confidence interval of mean	
					Lower	Upper
0-2	3DV	68	0.21	0.26	0.14	0.27
	X		0.04	0.20	-0.01	0.09
	Y		0.04	0.16	0.00	0.08
	Z		-0.02	0.20	-0.07	0.03
2-4	3DV	113	0.27	0.22	0.23	0.31
	X		0.04	0.16	0.01	0.07
	Y		0.05	0.24	0.01	0.10
	Z		-0.05	0.18	-0.09	-0.02
4-6	3DV	87	0.43	0.42	0.34	0.52
	X		0.02	0.27	-0.04	0.08
	Y		0.05	0.33	-0.02	0.12
	Z		-0.16	0.39	-0.24	-0.07
6-8	3DV	72	0.51	0.35	0.42	0.59
	X		0.10	0.37	0.02	0.19
	Y		0.04	0.33	-0.04	0.11
	Z		-0.14	0.33	-0.22	-0.07
8-10	3DV	58	0.46	0.29	0.38	0.53
	X		0.03	0.35	-0.06	0.12
	Y		0.13	0.25	0.06	0.19
	Z		-0.21	0.21	-0.26	-0.15
10-12	3DV	33	0.53	0.38	0.39	0.66

	X		0.05	0.28	-0.04	0.15
	Y		0.21	0.37	0.08	0.34
	Z		-0.09	0.40	-0.23	0.05
>12	3DV	22	0.48	0.31	0.35	0.62
	X		0.19	0.26	0.08	0.30
	Y		0.21	0.29	0.08	0.33
	Z		-0.12	0.31	-0.26	0.02

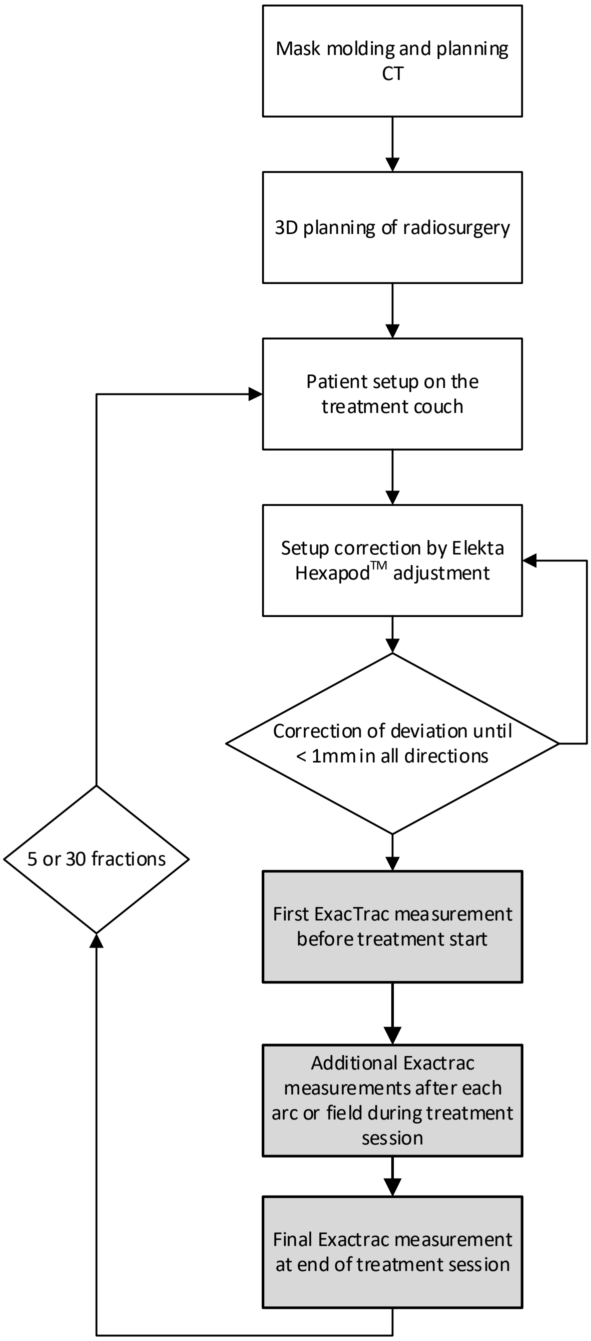

Supplementary Figure 1: Patient setup and repeated intrafractional tracking of head movement
